# [1-(Anthracen-9-ylmeth­yl)-1,4,7,10-tetra­aza­cyclododeca­ne]chlorido­zinc(II) nitrate

**DOI:** 10.1107/S2414314624006655

**Published:** 2024-07-12

**Authors:** Yoshimi Ichimaru, Kirara Sugiura, Koichi Kato, Yuki Kondo, Masaaki Kurihara, Wanchun Jin, Masanori Imai, Hiromasa Kurosaki

**Affiliations:** a Faculty of Pharmaceutical Sciences, Shonan University of Medical Sciences, 16-48, Kamishinano, Totsuka-ku, Yokohama, Kanagawa, 244-0806, Japan; b College of Pharmacy Kinjo Gakuin University, 2-1723 Omori Moriyamaku Nagoya Aichi 463-8521 Japan; Vienna University of Technology, Austria

**Keywords:** crystal structure, cyclen, [12]aneN_4_, anthracene, T-shaped π inter­actions

## Abstract

In the title salt, [ZnCl(C_23_H_30_N_4_)]NO_3_, the central Zn^II^ atom of the complex cation is coordinated in a square-pyramidal arrangement by four nitro­gen atoms from cyclen (1,4,7,10-tetra­aza­cyclo­dodeca­ne) in the basal plane and one chlorido ligand in the apical position. The anthracene group attached to cyclen contributes to the crystal packing through inter­molecular T-shaped π inter­actions. Additionally, the nitrate anion participates in inter­molecular N—H⋯O hydrogen bonds with cyclen.

## Abstract 2

The Zn^II^ atom in the complex cation of the title salt has a square-pyramidal coordination environment defined by four nitro­gen atoms from cyclen (1,4,7,10-tetra­aza­cyclo­dodeca­ne) in the basal plane and one chlorido ligand in the apical position.

## Graphical abstract

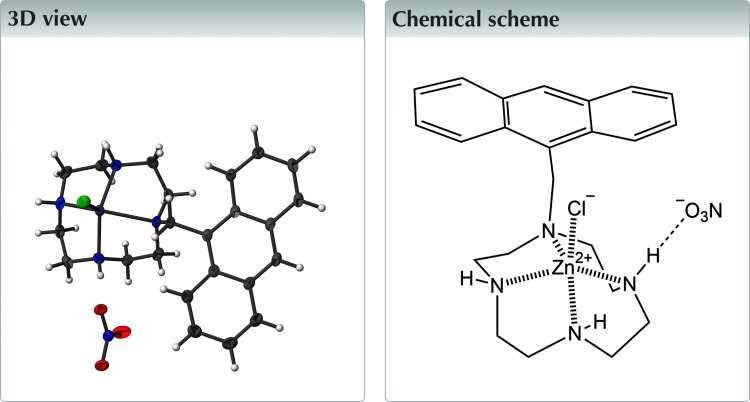

## Structure description

Complexes of 1,4,7,10-tetra­aza­cyclo­dodecane (cyclen or [12]aneN_4_) derivatives with Zn^II^ have been used as biological probes to elucidate the intrinsic roles of Zn^II^ in enzyme models such as phosphatase, alcohol de­hydrogenase, and β-lactamase (Koike & Kimura, 1991 ▸; Koike *et al.*, 1994 ▸; Kimura *et al.*, 1992 ▸). Cyclen conjugated with the anthracenyl methyl group, 1-(anthracen-9-ylmeth­yl)-1,4,7,10-tetra­aza­cyclo­dodecane, has been developed as a fluorescent chemosensor for detecting pH and transition-metal cations in aqueous solution (Akkaya *et al.*, 1990 ▸; Huston *et al.*, 1990 ▸). In this context, we present the crystal structure of the title salt, [ZnCl(C_23_H_30_N_4_)]NO_3_.

**Figure 1:**
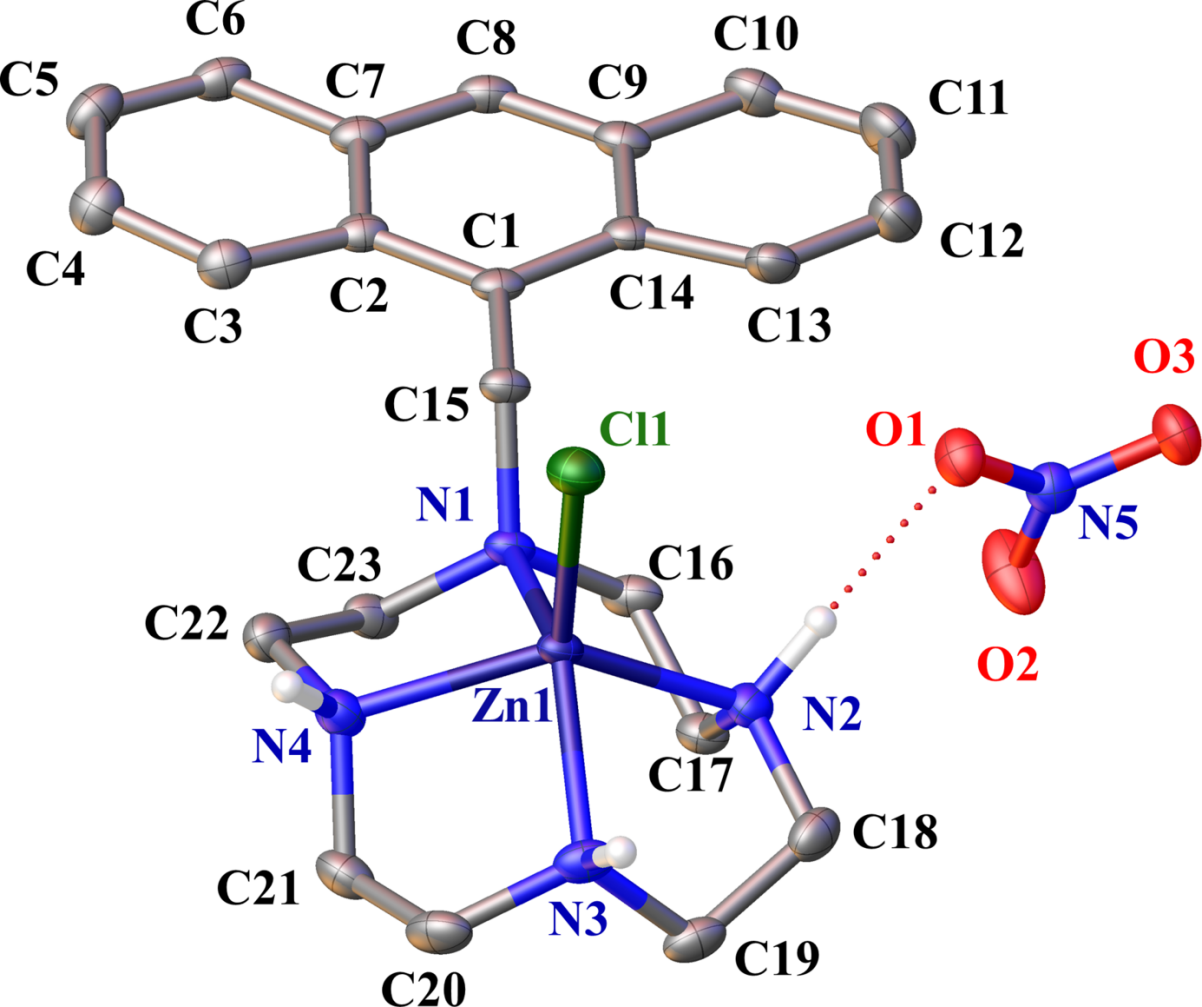
The mol­ecular structures of the complex cation and the anion in the title salt with displacement ellipsoids drawn at the 50% probability level. C-bound H atoms are omitted for clarity; the hydrogen bond is represented as a red dotted line.

**Figure 2:**
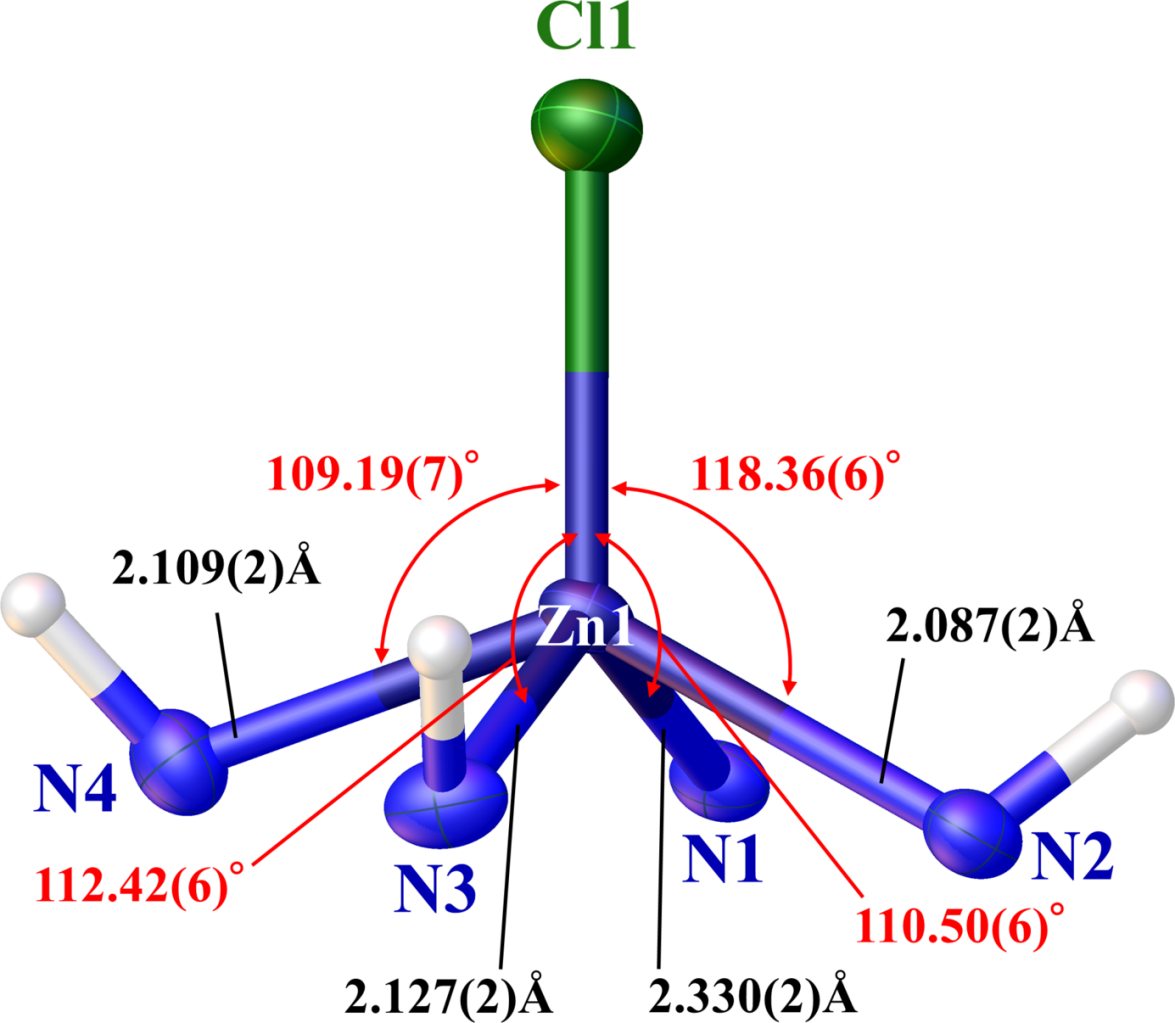
The coordination polyhedron around Zn1, with displacement ellipsoids drawn at the 50% probability level. Bond angles are depicted in red, whereas bond lengths are shown in black.

The crystal structure of the title compound comprises a [Zn(C_23_H_30_N_4_)Cl]^+^ complex cation and a nitrate anion (Fig. 1 ▸). The coordination environment around the Zn^II^ atom is slightly distorted square-pyramidal, with the coordination geometry index (Addison *et al.*, 1984 ▸), τ = (*β* − *α*) / 60° = 0.08, where *α* [132.23 (9)°] and *β* [136.98 (8)°] are the second-largest and largest angles around the central Zn^II^ atom, respectively. A τ value of 0 corresponds to an ideal square pyramid, while a value of 1 corresponds to an ideal trigonal bipyramid. The four nitro­gen atoms N1, N2, N3, and N4 of cyclen form the basal plane, with the chlorido ligand occupying the apical position. The mean Zn1—N bond length of 2.16 Å (Fig. 2 ▸) is comparable to that (2.13 Å) observed in the crystal structure of the salt Zn(C_23_H_30_N_4_)]^+^(ClO_4_)^2−^ (Ichimaru *et al.*, 2021 ▸). The Zn^II^ atom is displaced by 0.8306 (12) Å above the mean basal plane toward the apical chlorido ligand. The Zn—Cl bond length of 2.2464 (7) Å is comparable to that found in other Zn^II^–polyamine complexes with chlorido ligands, such as chlorido­(1,4,7,11-tetra­aza­cyclo­tetra­decane-*N*,*N′*,*N′′*,*N′′′*)zinc(II) perchlorate [2.2734 (8) Å; Lu *et al.*, 1997 ▸] or bis­[μ-chlorido-(1,4,8,11-tetra­cyclo­tetra­deca­ne)zinc(II)] tetra­chlor­ido­zincate(II) hemihydrate [2.288 (5) Å; Alcock *et al.*, 1992 ▸]. The presence of Cl^−^ as a ligand can be deduced from the synthesis conditions (see *Synthesis and crystallization*). The bromine salt of the ligand was freed by an anion-exchange resin. In this process, hydro­chloric acid was employed to regenerate the resin to its chloride anion form, which is the source of Cl^−^ binding to the Zn^II^ atom.

**Figure 3:**
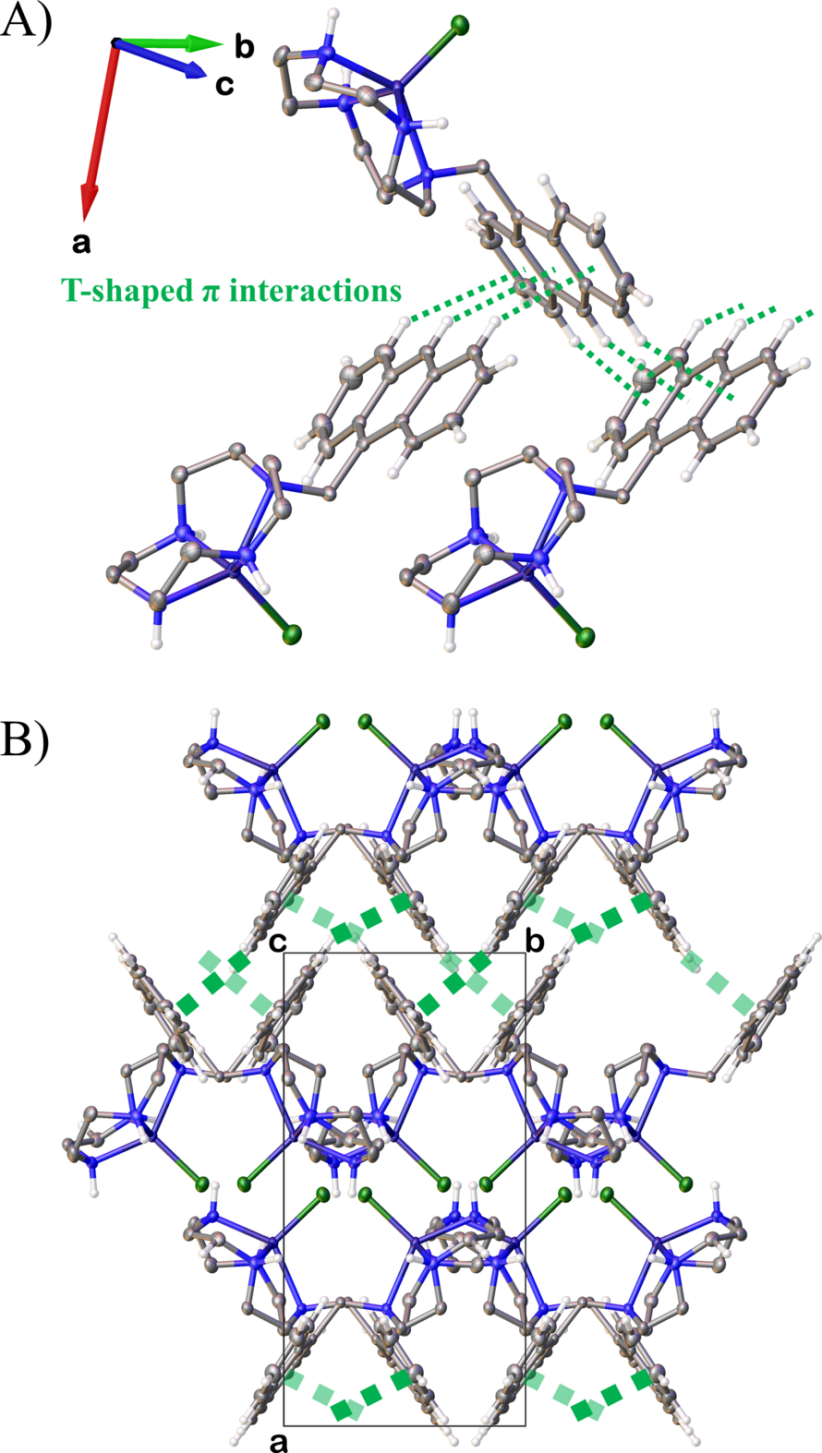
A schematic drawing of the T-shaped π–π inter­actions, with displacement ellipsoids drawn at the 50% probability level. Methyl­ene H atoms of cyclen rings and nitrate ions were omitted for clarity; T-shaped π–π inter­actions are depicted as green dotted lines.

The anthracene group exhibits a slight deviation from planarity, with fold angles of 4.69 (10)° between the *A* (C2–C7) and *B* (C1, C2, C7, C8, C9, C14) rings and 2.78 (11)° between the *B* and *C* (C9–C14) rings. The torsion angle defined by Zn1—N1—C15—C1 is 170.33 (18)°, positioning the anthracene group away from the macrocyclic ring, thereby preventing repulsive inter­actions with the Cl atom. In the crystal, nitrate O1 forms inter­molecular hydrogen bonds with H2 of the Zn^II^ complex and H3 of a neighboring mol­ecule. The hydrogen-bond distances O1⋯H2 and O1^i^⋯H3 are 1.985 and 2.16 Å (Table 1 ▸). These inter­actions contribute to the formation of a spiral structure extending parallel to the *b* axis direction of the crystal. Additionally, inter­molecular T-shaped π inter­actions (Jin *et al.*, 2022 ▸) occur between the anthracene ring and a neighboring anthracene ring [symmetry code: (ii): −*x*, 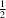 + *y*, 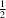 − *z*] (Fig. 3 ▸). The distance between H8 and the centroid (*Cg*) of the middle ring of the neighboring anthracene ring is 2.96 Å, and the angle C8—H8⋯*Cg* is 152°.

## Synthesis and crystallization

Under a nitro­gen atmosphere, 9-chloro­methyl­anthracene (2.40 g, 10.6 mmol) and 1,4,7-tris­(*tert*-butyl­oxycarbon­yl)-1,4,7,10-tetra­aza­cyclo­dodecane (3Boc-cyclen) (5.0 g, 10.6 mmol) (Kimura *et al.*, 1997 ▸) were dissolved in a mixture of aceto­nitrile (130 ml) and DMF (40 ml) and stirred at 373 K for 18 h in the presence of Na_2_CO_3_ (2.20 g, 12.1 mmol). After the reaction, CH_2_Cl_2_ (150 ml) was added to the reaction solution and extracted, the organic layer was washed with water (200 ml × 3) and dried with anhydrous Na_2_SO_4_, and the organic solvent was removed *in vacuo* to obtain the crude product. The residue was purified by silica gel column chromatography (3% MeOH–CH_2_Cl_2_) to obtain *N*-(9-anthra­cenylmeth­yl)-*N′*,*N′′*,*N′′′-*tris­(*tert*-butyl­oxycarbon­yl)-1,4,7,10-tetra­aza­cyclo­dodecane, *N*-Ant-(3Boc-cyclen), as a yellow solid (3.27 g, 47%). To an EtOH solution (30 ml) of *N*-Ant-(3Boc-cyclen) (1.00 g, 1.5 mmol), aqueous HBr (47%_wt_, 6 ml) was added and stirred at 273 K overnight. The resulting mixture was concentrated *in vacuo* below 308 K. The obtained residue was dissolved in water (2 ml) and washed with Et_2_O (10 ml × 3). Then, the aqueous layer was evaporated to dryness. The residue was neutralized by anion-exchange resin (Amberlite IRA-400, OH^−^ form), and the eluant was evaporated to obtain the desired ligand, *N*-Ant-cyclen, as a yellow amorphous solid (287 mg, 53%).

The title complex was prepared by adding a MeOH solution (1 ml) of Zn(NO_3_)_2_·6H_2_O (235 mg, 0.8 mmol) to a MeOH solution (5 ml) of *N*-Ant-cyclen (287 mg, 0.8 mmol). The mixture was heated, with stirring, at 323 K for 2 h and then concentrated. After the resulting residue was dissolved in a MeOH–water mixture (*v*/*v* = 1/1; 2 ml each) and filtrated, the filtrate was allowed to stand for 10 days at room temperature to obtain the title salt (286 mg, 84%).

## Refinement

Crystal data, data collection and structure refinement details are summarized in Table 2 ▸.

## Supplementary Material

Crystal structure: contains datablock(s) I. DOI: 10.1107/S2414314624006655/wm4217sup1.cif

Structure factors: contains datablock(s) I. DOI: 10.1107/S2414314624006655/wm4217Isup2.hkl

CCDC reference: 2368358

Additional supporting information:  crystallographic information; 3D view; checkCIF report

## Figures and Tables

**Table 1:** Hydrogen-bond geometry (Å, °)

*D*—H⋯*A*	*D*—H	H⋯*A*	*D*⋯*A*	*D*—H⋯*A*
N2—H2⋯O1	1.00	1.98	2.983 (3)	175
N3—H3⋯O1^i^	1.00	2.16	3.025 (3)	144

Symmetry code: (i) 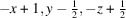.

**Table 2:** Experimental details

Crystal data	
Chemical formula	[ZnCl(C_23_H_30_N_4_)]NO_3_
*M*_r_	525.34
Crystal system, space group	Monoclinic, *P*2_1_/*c*
Temperature (K)	93
*a*, *b*, *c* (Å)	15.9086 (1), 7.8088 (1), 19.5342 (2)
β (°)	106.157 (1)
*V* (Å^3^)	2330.83 (4)
*Z*	4
Radiation type	Cu *K*α
μ (mm^−1^)	2.81
Crystal size (mm)	0.35 × 0.25 × 0.12

Data collection	
Diffractometer	Rigaku XtaLAB Synergy-i
Absorption correction	Multi-scan (*CrysAlis PRO*; Rigaku OD, 2022 ▸)
*T*_min_, *T*_max_	0.619, 1.000
No. of measured, independent and observed [*I* > 2σ(*I*)] reflections	21217, 4271, 4097
*R*_int_	0.027
(sin θ/λ)_max_ (Å^−1^)	0.603

Refinement	
*R*[*F*^2^ > 2σ(*F*^2^)], *wR*(*F*^2^), *S*	0.041, 0.110, 1.05
No. of reflections	4271
No. of parameters	298
H-atom treatment	H-atom parameters constrained
Δρ_max_, Δρ_min_ (e Å^−3^)	1.65, −0.68

Computer programs: *CrysAlis PRO* (Rigaku OD, 2022 ▸), *SHELXT* (Sheldrick, 2015*a* ▸), *SHELXL* (Sheldrick, 2015*b* ▸), *OLEX2* (Dolomanov *et al.*, 2009 ▸) and *publCIF* (Westrip, 2010 ▸).

## full crystallographic data

### [1-(Anthracen-9-ylmethyl)-1,4,7,10-tetraazacyclododecane]chloridozinc(II) nitrate Crystal data

**Table:**

C_23_H_30_ClN_4_Zn^+^·NO_3_^−^	*F*(000) = 1096
*M**_r_* = 525.34	*D*_x_ = 1.497 Mg m^−^^3^
Monoclinic, *P*2_1_/*c*	Cu *K*α radiation, λ = 1.54184 Å
*a* = 15.9086 (1) Å	Cell parameters from 16543 reflections
*b* = 7.8088 (1) Å	θ = 2.4–68.3°
*c* = 19.5342 (2) Å	µ = 2.81 mm^−^^1^
β = 106.157 (1)°	*T* = 93 K
*V* = 2330.83 (4) Å^3^	Plate, yellow
*Z* = 4	0.35 × 0.25 × 0.12 mm

### [1-(Anthracen-9-ylmethyl)-1,4,7,10-tetraazacyclododecane]chloridozinc(II) nitrate Data collection

**Table:**

Rigaku XtaLAB Synergy-i diffractometer	4097 reflections with *I* > 2σ(*I*)
Detector resolution: 10.0 pixels mm^-1^	*R*_int_ = 0.027
ω scans	θ_max_ = 68.4°, θ_min_ = 2.9°
Absorption correction: multi-scan (CrysAlisPro; Rigaku OD, 2022)	*h* = −19→19
*T*_min_ = 0.619, *T*_max_ = 1.000	*k* = −9→9
21217 measured reflections	*l* = −23→22
4271 independent reflections	

### [1-(Anthracen-9-ylmethyl)-1,4,7,10-tetraazacyclododecane]chloridozinc(II) nitrate Refinement

**Table:**

Refinement on *F*^2^	0 restraints
Least-squares matrix: full	Hydrogen site location: inferred from neighbouring sites
*R*[*F*^2^ > 2σ(*F*^2^)] = 0.041	H-atom parameters constrained
*wR*(*F*^2^) = 0.110	*w* = 1/[σ^2^(*F*_o_^2^) + (0.0537*P*)^2^ + 5.0389*P*] where *P* = (*F*_o_^2^ + 2*F*_c_^2^)/3
*S* = 1.05	(Δ/σ)_max_ = 0.001
4271 reflections	Δρ_max_ = 1.65 e Å^−^^3^
298 parameters	Δρ_min_ = −0.67 e Å^−^^3^

### [1-(Anthracen-9-ylmethyl)-1,4,7,10-tetraazacyclododecane]chloridozinc(II) nitrate Special details

**Table:**

Geometry. All esds (except the esd in the dihedral angle between two l.s. planes) are estimated using the full covariance matrix. The cell esds are taken into account individually in the estimation of esds in distances, angles and torsion angles; correlations between esds in cell parameters are only used when they are defined by crystal symmetry. An approximate (isotropic) treatment of cell esds is used for estimating esds involving l.s. planes.

### [1-(Anthracen-9-ylmethyl)-1,4,7,10-tetraazacyclododecane]chloridozinc(II) nitrate Fractional atomic coordinates and isotropic or equivalent isotropic displacement parameters (Å^2^)

**Table:**

	*x*	*y*	*z*	*U*_iso_*/*U*_eq_	
Zn1	0.38731 (2)	0.45659 (4)	0.35923 (2)	0.01650 (12)	
Cl1	0.48909 (4)	0.66348 (8)	0.38926 (4)	0.02646 (17)	
O1	0.38618 (12)	0.6627 (3)	0.16411 (10)	0.0250 (4)	
O2	0.35799 (13)	0.6797 (3)	0.04891 (10)	0.0283 (4)	
N1	0.24737 (14)	0.5751 (3)	0.32627 (12)	0.0179 (4)	
N2	0.34428 (15)	0.3815 (3)	0.25262 (12)	0.0210 (5)	
H2	0.354759	0.475940	0.221385	0.025*	
N3	0.44297 (14)	0.2069 (3)	0.37340 (13)	0.0234 (5)	
H3	0.507715	0.216524	0.381655	0.028*	
O3	0.27592 (15)	0.5258 (4)	0.09680 (12)	0.0425 (6)	
N4	0.34614 (15)	0.3897 (3)	0.44943 (12)	0.0233 (5)	
H4	0.393328	0.423402	0.492937	0.028*	
N5	0.33966 (14)	0.6225 (3)	0.10271 (12)	0.0223 (5)	
C1	0.18291 (16)	0.8823 (3)	0.30190 (14)	0.0178 (5)	
C2	0.13492 (16)	0.9487 (3)	0.34735 (14)	0.0183 (5)	
C7	0.06198 (17)	1.0620 (3)	0.31847 (15)	0.0194 (5)	
C17	0.24931 (17)	0.3503 (3)	0.23785 (15)	0.0213 (5)	
H17A	0.238613	0.257067	0.268744	0.026*	
H17B	0.224518	0.315513	0.187516	0.026*	
C15	0.26134 (16)	0.7645 (3)	0.33143 (14)	0.0173 (5)	
H15A	0.306094	0.792998	0.306741	0.021*	
H15B	0.286579	0.793243	0.382389	0.021*	
C8	0.03866 (17)	1.1046 (3)	0.24673 (15)	0.0209 (5)	
H8	−0.010901	1.175629	0.227958	0.025*	
C9	0.08644 (17)	1.0455 (3)	0.20166 (15)	0.0205 (5)	
C14	0.16158 (17)	0.9372 (3)	0.22978 (14)	0.0192 (5)	
C6	0.01563 (17)	1.1335 (3)	0.36509 (15)	0.0234 (6)	
H6	−0.033418	1.205501	0.345867	0.028*	
C16	0.20644 (18)	0.5133 (4)	0.25238 (15)	0.0236 (6)	
H16A	0.212198	0.602408	0.217920	0.028*	
H16B	0.143295	0.492520	0.245818	0.028*	
C10	0.06288 (19)	1.0952 (4)	0.12807 (15)	0.0270 (6)	
H10	0.012570	1.164427	0.109607	0.032*	
C13	0.21288 (19)	0.8961 (3)	0.18199 (15)	0.0241 (6)	
H13	0.265276	0.832090	0.199311	0.029*	
C3	0.15766 (17)	0.9180 (4)	0.42258 (14)	0.0218 (5)	
H3A	0.205360	0.844130	0.443490	0.026*	
C5	0.04022 (18)	1.1008 (4)	0.43543 (16)	0.0267 (6)	
H5	0.009087	1.150559	0.465472	0.032*	
C19	0.40575 (18)	0.1105 (4)	0.30722 (16)	0.0257 (6)	
H19A	0.348153	0.062604	0.307287	0.031*	
H19B	0.444945	0.014239	0.303947	0.031*	
C4	0.11287 (18)	0.9915 (4)	0.46489 (15)	0.0262 (6)	
H4A	0.130287	0.969705	0.514699	0.031*	
C20	0.4252 (2)	0.1301 (4)	0.43712 (16)	0.0287 (6)	
H20A	0.474231	0.155658	0.479727	0.034*	
H20B	0.420519	0.004167	0.431476	0.034*	
C21	0.34052 (19)	0.2010 (4)	0.44747 (16)	0.0276 (6)	
H21A	0.290232	0.163987	0.407747	0.033*	
H21B	0.331815	0.157511	0.492607	0.033*	
C18	0.39519 (19)	0.2283 (4)	0.24406 (16)	0.0270 (6)	
H18A	0.453501	0.263582	0.240278	0.032*	
H18B	0.364569	0.167201	0.199724	0.032*	
C22	0.26506 (19)	0.4805 (4)	0.45083 (15)	0.0253 (6)	
H22A	0.280205	0.594958	0.472524	0.030*	
H22B	0.235127	0.415636	0.480764	0.030*	
C23	0.20380 (17)	0.5006 (4)	0.37670 (16)	0.0233 (6)	
H23A	0.179688	0.387098	0.358880	0.028*	
H23B	0.154274	0.575060	0.378989	0.028*	
C12	0.1885 (2)	0.9464 (4)	0.11281 (17)	0.0316 (7)	
H12	0.223574	0.915233	0.082604	0.038*	
C11	0.1111 (2)	1.0449 (4)	0.08451 (17)	0.0326 (7)	
H11	0.093660	1.075128	0.035468	0.039*	

### [1-(Anthracen-9-ylmethyl)-1,4,7,10-tetraazacyclododecane]chloridozinc(II) nitrate Atomic displacement parameters (Å^2^)

**Table:**

	*U*^11^	*U*^22^	*U*^33^	*U*^12^	*U*^13^	*U*^23^
Zn1	0.01667 (19)	0.01153 (19)	0.0223 (2)	−0.00095 (12)	0.00709 (14)	0.00115 (12)
Cl1	0.0282 (3)	0.0217 (3)	0.0285 (3)	−0.0034 (3)	0.0061 (3)	0.0003 (3)
O1	0.0227 (9)	0.0308 (11)	0.0192 (9)	−0.0042 (8)	0.0023 (8)	−0.0008 (8)
O2	0.0308 (11)	0.0339 (11)	0.0207 (10)	−0.0004 (9)	0.0078 (8)	0.0065 (8)
N1	0.0166 (10)	0.0125 (10)	0.0242 (11)	0.0007 (8)	0.0053 (9)	0.0022 (9)
N2	0.0226 (11)	0.0188 (11)	0.0233 (11)	−0.0027 (9)	0.0092 (9)	0.0002 (9)
N3	0.0175 (11)	0.0171 (11)	0.0329 (13)	0.0024 (9)	0.0026 (9)	−0.0025 (10)
O3	0.0292 (12)	0.0666 (17)	0.0279 (11)	−0.0246 (11)	0.0017 (9)	0.0031 (11)
N4	0.0245 (12)	0.0216 (12)	0.0244 (12)	0.0023 (10)	0.0079 (9)	0.0047 (9)
N5	0.0195 (11)	0.0242 (12)	0.0229 (12)	0.0011 (9)	0.0052 (9)	0.0005 (9)
C1	0.0161 (12)	0.0106 (11)	0.0259 (13)	−0.0026 (9)	0.0043 (10)	−0.0008 (10)
C2	0.0144 (12)	0.0138 (12)	0.0253 (13)	−0.0024 (9)	0.0033 (10)	−0.0015 (10)
C7	0.0154 (12)	0.0138 (12)	0.0272 (14)	−0.0021 (10)	0.0027 (10)	−0.0019 (10)
C17	0.0224 (13)	0.0163 (13)	0.0246 (13)	−0.0023 (10)	0.0055 (11)	0.0013 (10)
C15	0.0152 (11)	0.0123 (12)	0.0246 (13)	0.0003 (9)	0.0056 (10)	0.0015 (10)
C8	0.0165 (12)	0.0133 (12)	0.0309 (14)	0.0004 (10)	0.0029 (10)	0.0015 (11)
C9	0.0190 (13)	0.0137 (12)	0.0267 (14)	−0.0025 (10)	0.0030 (11)	0.0026 (10)
C14	0.0195 (12)	0.0109 (11)	0.0268 (14)	−0.0032 (10)	0.0060 (11)	−0.0005 (10)
C6	0.0176 (12)	0.0173 (13)	0.0343 (15)	0.0012 (10)	0.0056 (11)	−0.0037 (11)
C16	0.0223 (13)	0.0164 (13)	0.0277 (14)	0.0004 (11)	−0.0002 (11)	0.0010 (11)
C10	0.0258 (14)	0.0223 (14)	0.0309 (15)	0.0005 (11)	0.0047 (12)	0.0077 (12)
C13	0.0289 (14)	0.0153 (13)	0.0300 (15)	0.0029 (11)	0.0111 (12)	0.0036 (11)
C3	0.0157 (12)	0.0226 (13)	0.0249 (14)	0.0010 (10)	0.0022 (10)	−0.0006 (11)
C5	0.0208 (13)	0.0277 (15)	0.0324 (15)	0.0011 (12)	0.0084 (11)	−0.0086 (12)
C19	0.0216 (13)	0.0184 (13)	0.0363 (16)	0.0023 (11)	0.0066 (12)	−0.0066 (12)
C4	0.0215 (14)	0.0308 (15)	0.0244 (14)	0.0004 (12)	0.0032 (11)	−0.0030 (12)
C20	0.0331 (15)	0.0173 (13)	0.0297 (15)	0.0034 (12)	−0.0012 (12)	0.0033 (11)
C21	0.0303 (15)	0.0228 (14)	0.0285 (15)	−0.0033 (12)	0.0062 (12)	0.0079 (12)
C18	0.0262 (14)	0.0263 (15)	0.0327 (15)	−0.0018 (12)	0.0151 (12)	−0.0081 (12)
C22	0.0295 (15)	0.0220 (14)	0.0284 (15)	−0.0003 (11)	0.0146 (12)	0.0009 (11)
C23	0.0193 (13)	0.0188 (13)	0.0356 (15)	−0.0001 (11)	0.0138 (12)	0.0035 (12)
C12	0.0414 (18)	0.0260 (15)	0.0320 (16)	0.0036 (13)	0.0177 (14)	0.0051 (12)
C11	0.0415 (18)	0.0297 (16)	0.0260 (15)	0.0025 (13)	0.0084 (13)	0.0092 (12)

### [1-(Anthracen-9-ylmethyl)-1,4,7,10-tetraazacyclododecane]chloridozinc(II) nitrate Geometric parameters (Å, º)

**Table:**

Zn1—Cl1	2.2466 (7)	C9—C14	1.442 (4)
Zn1—N1	2.330 (2)	C9—C10	1.435 (4)
Zn1—N2	2.087 (2)	C14—C13	1.437 (4)
Zn1—N3	2.127 (2)	C6—H6	0.9500
Zn1—N4	2.109 (2)	C6—C5	1.344 (4)
O1—N5	1.261 (3)	C16—H16A	0.9900
O2—N5	1.249 (3)	C16—H16B	0.9900
N1—C15	1.495 (3)	C10—H10	0.9500
N1—C16	1.489 (3)	C10—C11	1.353 (4)
N1—C23	1.473 (3)	C13—H13	0.9500
N2—H2	1.0000	C13—C12	1.356 (4)
N2—C17	1.477 (3)	C3—H3A	0.9500
N2—C18	1.480 (4)	C3—C4	1.359 (4)
N3—H3	1.0000	C5—H5	0.9500
N3—C19	1.471 (4)	C5—C4	1.423 (4)
N3—C20	1.478 (4)	C19—H19A	0.9900
O3—N5	1.244 (3)	C19—H19B	0.9900
N4—H4	1.0000	C19—C18	1.510 (4)
N4—C21	1.476 (4)	C4—H4A	0.9500
N4—C22	1.479 (4)	C20—H20A	0.9900
C1—C2	1.420 (4)	C20—H20B	0.9900
C1—C15	1.528 (3)	C20—C21	1.521 (4)
C1—C14	1.420 (4)	C21—H21A	0.9900
C2—C7	1.443 (4)	C21—H21B	0.9900
C2—C3	1.433 (4)	C18—H18A	0.9900
C7—C8	1.387 (4)	C18—H18B	0.9900
C7—C6	1.435 (4)	C22—H22A	0.9900
C17—H17A	0.9900	C22—H22B	0.9900
C17—H17B	0.9900	C22—C23	1.512 (4)
C17—C16	1.508 (4)	C23—H23A	0.9900
C15—H15A	0.9900	C23—H23B	0.9900
C15—H15B	0.9900	C12—H12	0.9500
C8—H8	0.9500	C12—C11	1.426 (4)
C8—C9	1.392 (4)	C11—H11	0.9500
			
Cl1—Zn1—N1	110.50 (6)	C7—C6—H6	119.3
N2—Zn1—Cl1	118.36 (6)	C5—C6—C7	121.3 (3)
N2—Zn1—N1	79.22 (8)	C5—C6—H6	119.3
N2—Zn1—N3	83.20 (9)	N1—C16—C17	110.9 (2)
N2—Zn1—N4	132.23 (9)	N1—C16—H16A	109.4
N3—Zn1—Cl1	112.42 (6)	N1—C16—H16B	109.5
N3—Zn1—N1	136.98 (8)	C17—C16—H16A	109.4
N4—Zn1—Cl1	109.19 (7)	C17—C16—H16B	109.4
N4—Zn1—N1	80.46 (8)	H16A—C16—H16B	108.0
N4—Zn1—N3	83.05 (9)	C9—C10—H10	119.5
C15—N1—Zn1	105.17 (14)	C11—C10—C9	121.1 (3)
C16—N1—Zn1	105.40 (15)	C11—C10—H10	119.5
C16—N1—C15	113.8 (2)	C14—C13—H13	119.1
C23—N1—Zn1	104.45 (15)	C12—C13—C14	121.7 (3)
C23—N1—C15	115.8 (2)	C12—C13—H13	119.1
C23—N1—C16	111.1 (2)	C2—C3—H3A	119.1
Zn1—N2—H2	109.7	C4—C3—C2	121.8 (3)
C17—N2—Zn1	105.96 (16)	C4—C3—H3A	119.1
C17—N2—H2	109.7	C6—C5—H5	120.0
C17—N2—C18	114.0 (2)	C6—C5—C4	120.0 (3)
C18—N2—Zn1	107.57 (17)	C4—C5—H5	120.0
C18—N2—H2	109.7	N3—C19—H19A	109.8
Zn1—N3—H3	109.0	N3—C19—H19B	109.8
C19—N3—Zn1	107.67 (16)	N3—C19—C18	109.4 (2)
C19—N3—H3	109.0	H19A—C19—H19B	108.2
C19—N3—C20	114.1 (2)	C18—C19—H19A	109.8
C20—N3—Zn1	108.13 (17)	C18—C19—H19B	109.8
C20—N3—H3	109.0	C3—C4—C5	120.8 (3)
Zn1—N4—H4	108.1	C3—C4—H4A	119.6
C21—N4—Zn1	104.84 (17)	C5—C4—H4A	119.6
C21—N4—H4	108.1	N3—C20—H20A	109.6
C21—N4—C22	115.6 (2)	N3—C20—H20B	109.6
C22—N4—Zn1	111.83 (17)	N3—C20—C21	110.4 (2)
C22—N4—H4	108.1	H20A—C20—H20B	108.1
O2—N5—O1	119.9 (2)	C21—C20—H20A	109.6
O3—N5—O1	119.1 (2)	C21—C20—H20B	109.6
O3—N5—O2	121.0 (2)	N4—C21—C20	108.5 (2)
C2—C1—C15	120.5 (2)	N4—C21—H21A	110.0
C2—C1—C14	119.2 (2)	N4—C21—H21B	110.0
C14—C1—C15	120.0 (2)	C20—C21—H21A	110.0
C1—C2—C7	119.5 (2)	C20—C21—H21B	110.0
C1—C2—C3	123.6 (2)	H21A—C21—H21B	108.4
C3—C2—C7	116.7 (2)	N2—C18—C19	109.9 (2)
C8—C7—C2	120.1 (2)	N2—C18—H18A	109.7
C8—C7—C6	120.5 (2)	N2—C18—H18B	109.7
C6—C7—C2	119.3 (2)	C19—C18—H18A	109.7
N2—C17—H17A	110.0	C19—C18—H18B	109.7
N2—C17—H17B	110.0	H18A—C18—H18B	108.2
N2—C17—C16	108.4 (2)	N4—C22—H22A	109.3
H17A—C17—H17B	108.4	N4—C22—H22B	109.3
C16—C17—H17A	110.0	N4—C22—C23	111.4 (2)
C16—C17—H17B	110.0	H22A—C22—H22B	108.0
N1—C15—C1	118.7 (2)	C23—C22—H22A	109.3
N1—C15—H15A	107.6	C23—C22—H22B	109.3
N1—C15—H15B	107.6	N1—C23—C22	112.6 (2)
C1—C15—H15A	107.6	N1—C23—H23A	109.1
C1—C15—H15B	107.6	N1—C23—H23B	109.1
H15A—C15—H15B	107.1	C22—C23—H23A	109.1
C7—C8—H8	119.3	C22—C23—H23B	109.1
C7—C8—C9	121.3 (2)	H23A—C23—H23B	107.8
C9—C8—H8	119.3	C13—C12—H12	119.4
C8—C9—C14	119.6 (2)	C13—C12—C11	121.3 (3)
C8—C9—C10	120.5 (2)	C11—C12—H12	119.4
C10—C9—C14	119.9 (3)	C10—C11—C12	119.5 (3)
C1—C14—C9	119.9 (2)	C10—C11—H11	120.3
C1—C14—C13	123.7 (2)	C12—C11—H11	120.3
C13—C14—C9	116.4 (2)		
			
Zn1—N1—C15—C1	170.33 (18)	C15—C1—C2—C3	2.8 (4)
Zn1—N1—C16—C17	23.5 (2)	C15—C1—C14—C9	178.7 (2)
Zn1—N1—C23—C22	37.1 (2)	C15—C1—C14—C13	−3.6 (4)
Zn1—N2—C17—C16	58.9 (2)	C8—C7—C6—C5	−176.5 (3)
Zn1—N2—C18—C19	40.9 (2)	C8—C9—C14—C1	4.2 (4)
Zn1—N3—C19—C18	37.5 (2)	C8—C9—C14—C13	−173.7 (2)
Zn1—N3—C20—C21	29.3 (3)	C8—C9—C10—C11	176.9 (3)
Zn1—N4—C21—C20	49.6 (2)	C9—C14—C13—C12	−4.5 (4)
Zn1—N4—C22—C23	36.5 (3)	C9—C10—C11—C12	−2.3 (5)
N2—C17—C16—N1	−55.4 (3)	C14—C1—C2—C7	3.9 (4)
N3—C19—C18—N2	−53.5 (3)	C14—C1—C2—C3	−172.3 (2)
N3—C20—C21—N4	−54.3 (3)	C14—C1—C15—N1	−90.1 (3)
N4—C22—C23—N1	−51.2 (3)	C14—C9—C10—C11	−1.4 (4)
C1—C2—C7—C8	0.4 (4)	C14—C13—C12—C11	1.0 (5)
C1—C2—C7—C6	−177.8 (2)	C6—C7—C8—C9	175.7 (2)
C1—C2—C3—C4	176.3 (3)	C6—C5—C4—C3	−0.6 (5)
C1—C14—C13—C12	177.7 (3)	C16—N1—C15—C1	55.5 (3)
C2—C1—C15—N1	94.8 (3)	C16—N1—C23—C22	150.2 (2)
C2—C1—C14—C9	−6.2 (4)	C10—C9—C14—C1	−177.5 (2)
C2—C1—C14—C13	171.5 (2)	C10—C9—C14—C13	4.7 (4)
C2—C7—C8—C9	−2.5 (4)	C13—C12—C11—C10	2.6 (5)
C2—C7—C6—C5	1.8 (4)	C3—C2—C7—C8	176.9 (2)
C2—C3—C4—C5	1.0 (4)	C3—C2—C7—C6	−1.4 (4)
C7—C2—C3—C4	0.1 (4)	C19—N3—C20—C21	−90.4 (3)
C7—C8—C9—C14	0.2 (4)	C20—N3—C19—C18	157.5 (2)
C7—C8—C9—C10	−178.1 (2)	C21—N4—C22—C23	−83.4 (3)
C7—C6—C5—C4	−0.8 (4)	C18—N2—C17—C16	177.0 (2)
C17—N2—C18—C19	−76.3 (3)	C22—N4—C21—C20	173.2 (2)
C15—N1—C16—C17	138.2 (2)	C23—N1—C15—C1	−75.0 (3)
C15—N1—C23—C22	−78.0 (3)	C23—N1—C16—C17	−89.0 (3)
C15—C1—C2—C7	179.0 (2)		

### [1-(Anthracen-9-ylmethyl)-1,4,7,10-tetraazacyclododecane]chloridozinc(II) nitrate Hydrogen-bond geometry (Å, º)

**Table:**

*D*—H···*A*	*D*—H	H···*A*	*D*···*A*	*D*—H···*A*
N2—H2···O1	1.00	1.98	2.983 (3)	175
N3—H3···O1^i^	1.00	2.16	3.025 (3)	144

Symmetry code: (i) −*x*+1, *y*−1/2, −*z*+1/2.
